# Prognostic role of alpha-fetoprotein in patients with hepatocellular carcinoma treated with repeat transarterial chemoembolisation

**DOI:** 10.1186/s12885-020-06806-4

**Published:** 2020-05-29

**Authors:** Gauri Mishra, Anouk Dev, Eldho Paul, Wa Cheung, Jim Koukounaras, Ashu Jhamb, Ben Marginson, Beng Ghee Lim, Paul Simkin, Adina Borsaru, James Burnes, Mark Goodwin, Vivek Ramachandra, Manfred Spanger, John Lubel, Paul Gow, Siddharth Sood, Alexander Thompson, Marno Ryan, Amanda Nicoll, Sally Bell, Ammar Majeed, William Kemp, Stuart K. Roberts, The Melbourne Liver Group

**Affiliations:** 1 Gastroenterology, Monash Health, Melbourne, Australia; 2 School of Public Health and Preventive Medicine, Monash University, Melbourne, Australia; 3 Radiology, Alfred Health, Melbourne, Australia; 4 Radiology, St. Vincent’s Hospital, Melbourne, Australia; 5 Radiology, Royal Melbourne Hospital, Melbourne, Australia; 6 Radiology, Monash Health Eastern Health, Melbourne, Australia; 7 Radiology, Austin Health, Melbourne, Australia; 8 Radiology, Eastern Health, Melbourne, Australia; 9 Department of Gastroenterology, The Alfred Hospital, 55 Commercial Rd, Melbourne, 3004 Australia; 10 Gastroenterology, Austin Health, Melbourne, Australia; 11 Gastroenterology, Royal Melbourne Hospital, Melbourne, Australia; 12 Gastroenterology, St. Vincent’s Hospital, Melbourne, Australia; 13 Gastroenterology, Eastern Health, Melbourne, Australia

**Keywords:** Hepatocellular carcinoma, Repeat transarterial chemoembolisation, Prognosis, Alpha-fetoprotein

## Abstract

**Background:**

Repeat transarterial chemoembolisation (rTACE) is often required for hepatocellular carcinoma (HCC) to achieve disease control, however, current practice guidelines regarding treatment allocation vary significantly. This study aims to identify key factors associated with patient survival following rTACE to facilitate treatment allocation and prognostic discussion.

**Method:**

Patients with HCC undergoing rTACE at six Australian tertiary centers from 2009 to 2014 were included. Variables encompassing clinical, tumour, treatment type and response factors were analysed against the primary outcome of overall survival. Univariate analysis and multivariate Cox regression modelling were used to identify factors pre- and post-TACE therapy significantly associated with survival.

**Results:**

Total of 292 consecutive patients underwent rTACE with mainly Child Pugh A cirrhosis (61%) and BCLC stage A (57%) disease. Median overall survival (OS) was 30 months (IQR 15.2–50.2) from initial TACE. On multivariate analysis greater tumour number (*p* = 0.02), higher serum bilirubin (*p* = 0.007) post initial TACE, and hepatic decompensation (*p* = 0.001) post second TACE were associated with reduced survival. Patients with serum AFP ≥ 200 ng/ml following initial TACE had lower survival (*p* = 0.001), compared to patients with serum AFP level that remained < 200 ng/ml post-initial TACE, with an overall survival of 19.4 months versus 34.7 months (*p* = 0.0001) respectively.

**Conclusion:**

Serum AFP level following initial treatment in patients undergoing repeat TACE for HCC is a simple and useful clinical prognostic marker. Moreover, it has the potential to facilitate appropriate patient selection for rTACE particularly when used in conjunction with baseline tumour burden and severity of hepatic dysfunction post-initial TACE.

## Background

The majority of patients with unresectable HCC (uHCC) will undergo repeat transarterial chemoembolisation (TACE) therapy to optimize treatment response, however a significant proportion are at risk for an adverse outcome after repeat cycles due to either tumour progression or decline in hepatic reserve [1–4]. Whilst most international guidelines provide inclusion criteria for initial TACE, clinical criteria for eligibility for repeat TACE and factors predictive of poor outcomes are inadequately defined [5, 6]. In this context, several scoring systems have been developed to facilitate guidance in this area such as ART (Assessment for Retreatment with TACE) [7, 8] and ABCR (Alpha-fetoprotein, BCLC stage, Child-Pugh class, and radiological response) [9] score. However, their clinical utility has been limited in part due to their complexity and/or lack of applicability to the real world setting where *on demand* TACE is commonly employed and radiological response assessment has become more refined [10–13].

Therefore, further studies are required to identify key simple prognostic factors associated with overall survival (OS) following repeat TACE so as to improve patient selection and safety via the avoidance of unnecessary repeat procedures and unwanted side effects [6, 14]. Such prognostic data will facilitate clinician decision making and potentially improve patient survival as patients undergo timely stage migration to the next treatment option such as systemic therapy [15, 16].

In this study we analysed the prognostic factors associated with overall survival in patients undergoing repeat TACE and in particular the impact of the tumour marker alpha-fetoprotein (AFP). AFP is an established prognostic marker of both poorer HCC phenotype and more aggressive tumour biology [17–22]. While the optimum cut off value varies significantly in published literature on patients with HCC treated with TACE, it has been suggested that higher pre-treatment AFP is associated with earlier recurrence and poorer overall survival [23–25].

## Methods

### Study population

We retrospectively identified patients with HCC who had undergone TACE therapy from six tertiary centers in Melbourne, Australia between January 2009 and December 2014 using established HCC databases at each hospital and review of electronic medical records. All patients had the diagnosis of HCC confirmed on biopsy or established radiological criteria [26] and were deemed suitable for TACE after review by the multi-disciplinary team at the relevant hospital. Patients were included if they were classified as BCLC stage A, B, or C with relatively well preserved European Co-operative Oncology Group (ECOG) performance status of 0–2. Subjects who had undergone at least two cycles of TACE were included, with the exception of those who were administered TACE as a bridge to liver transplantation.

Pre- and post-TACE clinical, radiological and laboratory characteristics were recorded, including presence of cirrhosis based on biochemical and radiological criteria as previously described [16, 27]. Adverse events within 4 weeks of therapy were also recorded including hepatic decompensation as defined by the development of ascites, hepatic encephalopathy, hepatorenal syndrome or upper GI bleeding [28, 29]. Following approval of a low-risk application to the respective Institutional Ethics Committees, data regarding patient and tumour characteristics were collected for analysis, including clinical and radiological response, as defined by the mRECIST criteria [30, 31]. All patient data were de-identified prior to collation and statistical analysis.

### Primary outcome

Overall survival was calculated from the date of first TACE treatment to either date of death or last clinical follow-up, with censoring at 31st January 2019. The date of death was obtained from either the patient hospital records and MDT databases at each hospital or if missing from the Victorian Death and/or Cancer Registry.

### Statistical analysis

Continuous data were summarised using mean (standard deviation) or median (interquartile range) depending on the underlying distribution of the data. Categorical data were summarised using frequency tables, presenting the subject counts and percentages. Comparisons between groups (AFP < 200 versus ≥200 ng/ml) were made using the Student’s t-test for normally distributed continuous variables, Wilcoxon rank-sum test for non-normally distributed continuous variables and chi-square or Fisher’s exact test as appropriate for categorical variables.

The Kaplan-Meier product-limit method was used to plot survival as a function of time after treatment and to determine the median survival times. Comparisons between survival curves were made using the log-rank test. Univariate and multivariate analyses were performed via Cox proportional hazards regression to assess the effects of baseline clinical, liver disease, and tumour variables (pre-TACE 2) as well as tumour response variables (between TACE-1 and TACE-2) on overall survival.

Multivariate models were developed using a stepwise selection procedure and a backward elimination procedure before undergoing assessment for clinical and biological plausibility. Results from the Cox regression models were reported as hazard ratios (HR) and the corresponding 95% confidence intervals (95% CI). All reported *P*-values were two-sided with a *P* < 0.05 indicating statistical significance. Analyses were performed with the SAS software version 9.4 (SAS Institute, Cary, NC, USA).

## Results

### Patient characteristics

A total of 431 patients received TACE for HCC from 2009 to 2014 inclusive, of these 292 received at least two TACE treatments and were included in this study (Table 1). This cohort comprised mainly of BCLC stage A (57%) and B (39%) disease (Table 1). The majority of patients were male (87%), of Caucasian background (78%) and had predominantly alcohol (42%) or HCV (41%) related chronic liver disease. At baseline most had well compensated Child Pugh A (61%) cirrhosis with a smaller proportion having Child Pugh B disease (30%). Most patients had conventional TACE (cTACE) (79%) with the remainder receiving drug eluting beads TACE (DEB-TACE) (19%) or bland embolisation (TAE) (1%). Incomplete radiological response of the target lesion following initial TACE was common, as defined by mRECIST with majority having partial (43%), stable (9%) or progressive (8%) disease. Repeat TACE was provided on demand in all patients at a median interval of 2.5 months (IQR 1.4–7.7) following initial TACE, with a few individuals undergoing > 6 cycles. The most common complication of TACE observed was post-TACE syndrome (13%) with liver decompensation occurring in 1% of patients.

**Table 1:** Baseline characteristics of the cohort undergoing repeat TACE therapy

Baseline Characteristics	Overall cohort ***n*** = 292
Age (years), mean, (SD)	66 (10)
Male, n (%)	254 (87)
Female, n(%)	38 (13)
Ethnicity, n(%)	
Caucasian	229 (78)
Asian	51 (17)
Other	12 (4)
Aetiology of Liver disease, n(%)	
HCV / HBV / ETOH	120/58/122 (41/20/42)
NAFLD / Haemochromatosis/ other	67/12/14 (23/4/5)
BMI, mean (SD)	26 (24-30)
Serum markers, median (IQR)	
AFP, ng/ml	19 (5-175)
ALT, U/L	49 (32-78)
Albumin, g/L	34 (31-39)
Bilirubin, μmol/L	18 (12-27)
INR	1.1 (1.0-1.3)
Creatinine, μmol/L	75 (65-87)
Na, mmol/L	139 (137-140)
Liver function, n (%)	
Portal HTN / Ascites / HE	253/39/16 (87/13/5)
Child Pugh Score (A/B)	178/88/(61/30)
MELD score	9 (7-11)
ECOG (0/1), n (%)	127/165 (43/57)
Tumour Characteristics	
Tumour Nodules (1/2/3/>3), n (%)	127/65/20/80 (43/22/7/27)
Tumour Size, cm (median, IQR)	3.3 ( 2.0-5.0)
Macrovascular invasion, n (%)	9 (3)
Extrahepatic spread, n (%)	5 (2)
BCLC stage (A/B/C), n (%)	166/113/13 (57/39/4)
TACE treatments (2/3/>3) n (%)	132/80/80 (45/27/27)
Type (cTACE / DEB TACE / TAE)	232/56/3 (79/19/1)
Selectivity	
selective /superselective /non selective	197/48/43 (67/16/15)
mRECIST Response, n (%)	
Complete/Partial	69/127 (24/43)
Stable/Progressive	26/22 (9/8)
Adverse Events, n (%)	
Death	3 (1)
Post TACE syndrome/Decompensation	38/4 (13/1)
Renal dysfunction/other	3/12 (1/4)
Post TACE Treatment, n (%)	59 (20)
Resection/Ablation/PEI/SIRT	8/33/11/9 (3/11/4/3)

Serum AFP data was available in 260 (89%) patients prior to initial TACE, with the median baseline AFP level being 19 ng/ml (IQR 5–174.5). Of these patients, 135 (52%) had levels below 20 ng/ml, 60 patients (23%) had AFP ≥ 200 and 45 (17%) were ≥ 400 ng/ml prior to initial TACE. Following initial TACE, 110 patients (42%) had AFP of < 20 ng/ml, while 30 (12%) and 23 (9%) had AFP ≥ 200 ng/ml and ≥ 400 ng/ml respectively. In total, 177 (61%) of the overall cohort had AFP data available prior to both their first and second TACE for comparative analysis. Of these 30 patients (17%) had an AFP ≥ 200 ng/ml following initial TACE therapy and 147 (83%) had an AFP < 200 ng/ml.

### Overall survival

**Fig. 1:**
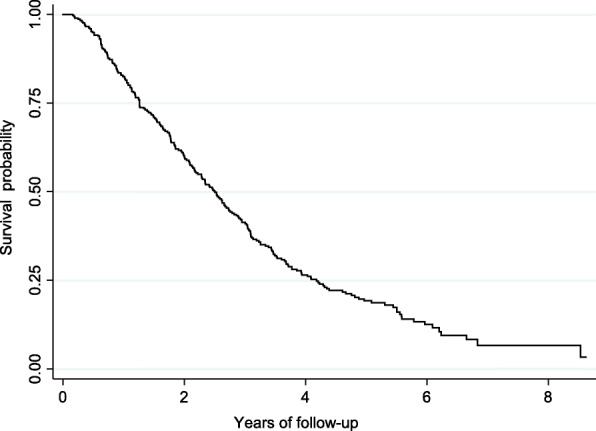
Kaplan Meier survival analysis of the overall cohort undergoing repeat TACE (*n* = 292)

During a median follow-up of 28 months (IQR 14.8–45.4) after the initial TACE, there were 82 (28%) patients who died. The median overall survival from time of first TACE therapy was 30 months (IQR 15.2–50.2) (Fig. 1).

### Predictors of overall survival

#### Univariate analysis

Univariate analysis compared all variables associated with OS encompassing hepatic synthetic function, tumour-related factors both pre- and post-initial TACE and radiological response (mRECIST). Baseline variables pre-initial TACE associated with improved survival in patients undergoing repeat TACE included single tumour (*p* = 0.043), BCLC stage A (*p* = 0.026) and higher serum albumin (*p* < 0.001) (Table 2a). In contrast, variables reflective of lower hepatic reserve prior to initial and repeat TACE including higher CP score (*p* < 0.001), serum bilirubin (*p* < 0.001), liver decompensation (*p* < 0.001) and ascites (*p* < 0.001) were significantly associated with reduced OS. Of note, renal dysfunction following the second TACE was associated with the highest risk of mortality [HR 3.46 (1.07–11.19), *p* = 0.035] (Table 2b).

**Table 2:** Univariate analysis of variables associated with overall survival in patients undergoing repeat TACE, with baseline variables (a) and subsequent to initial TACE (b)

	Overall cohort ***n*** = 292			
	Hazard Ratio	Lower 95% CI	Higher 95% CI	***P*** value
**Pre -TACE**				
**Variable**				
Single Tumour	0.76	0.59	1.00	0.043
Albumin, g/L	0.96	0.94	0.98	<0.001
Bilirubin, μmol/L	1.02	1.01	1.03	<0.001
Ascites	1.94	1.35	2.78	<0.001
Hepatic Encephalopathy	1.70	0.99	2.90	0.048
BCLC stage A	0.75	0.58	0.97	0.026
BCLC stage B	1.36	1.04	1.77	0.020
Child Pugh Score (5/6/7/8/9)	1.25	1.13	1.39	<0.001
AFP ≥ 200 ng/ml	1.48	1.07	2.04	0.015
**Post-TACE**				
**Variable**				
Post 1st TACE				
AFP ≥ 200 ng/ml	2.19	1.43	3.36	<0.001
Albumin, g/L	0.95	0.93	0.98	<0.001
Bilirubin, μmol/L	1.02	1.01	1.03	<0.001
Na, mmol/L	0.95	0.91	0.99	0.010
INR	1.70	1.12	2.59	0.012
MELD score	1.07	1.02	1.11	0.004
Child Pugh Score (5/6/7/8/9)	1.29	1.16	1.43	<0.001
Ascites	1.66	1.17	2.35	0.004
Post 2nd TACE				
Decompensation	3.39	1.85	6.21	<0.001
Renal dysfunction	3.46	1.07	11.19	0.035
Combination therapy	0.65	0.46	0.91	0.011
Ablation	0.56	0.35	0.88	0.011
Resection	0.27	0.10	0.76	0.011

Analysis of serum AFP as a continuous variable demonstrated a higher serum AFP following initial TACE was associated with lower survival (*p* < 0.001) in patients undergoing repeat TACE (data not shown). For increased interpretability of HR and CI the application of serum cut-off levels of 200 ng/ml and 400 ng/ml, demonstrated a significant relationship between both pre and post treatment serum AFP and overall survival (Table 3).

**Table 3:** Univariate analysis of pre and post initial TACE serum AFP levels and association with overall survival

Variable	n	%	Hazard Ratio	Lower 95% CI	Higher 95% CI	***P*** value
Baseline AFP ≥ 200 ng/ml	260	23%	1.48	1.07	2.04	0.015
Post initial TACE AFP ≥ 200 ng/ml	177	17%	2.19	1.43	3.36	<0.001
Baseline AFP ≥ 400 ng/ml	260	17%	1.36	0.95	1.95	0.088
Post Initial TACE AFP ≥ 400 ng/ml	177	13%	2.41	1.49	3.90	<0.001

#### Multivariate analysis

On multivariate analysis, factors significantly associated with lower survival in patients undergoing repeat TACE included an increase in number of tumour nodules at baseline (*p* = 0.02), a serum AFP ≥ 200 ng/ml (*p* = 0.001) and higher bilirubin following initial TACE (*p* = 0.007), and liver decompensation following repeat TACE (p = 0.001) (Table 4).

**Table 4:** Multivariate analysis of variables associated with overall survival in patients undergoing repeat TACE

Overall cohort ***n*** = 292				
Variable	Hazard Ratio	Lower 95% CI	Higher 95% CI	***P*** value
Baseline tumour number, 1, 2,3, > 3	1.18	1.03	1.36	0.020
Bilirubin post TACE 1	1.02	1.00	1.03	0.007
AFP ≥ 200 ng/ml post TACE 1	2.13	1.34	3.40	0.001
Decompensation post TACE 2	4.50	1.86	10.89	0.001

### Relationship between AFP and overall survival

**Fig. 2:**
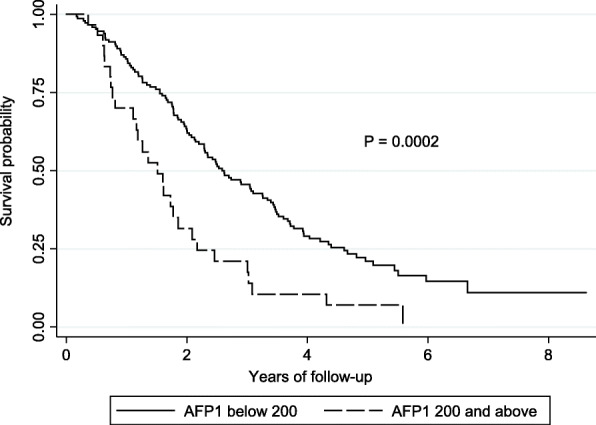
Kaplan Meier survival analysis of patients with an serum AFP above or below 200 ng/ml following initial TACE (AFP1)

On further subgroup analysis patients with a higher serum AFP ≥ 200 ng/ml following initial TACE had a significantly lower overall survival with median OS of 18.2 months (IQR 9.2 to 26.1) compared to those with serum AFP < 200 ng/ml of 31.1 months (IQR 18.7 to 55.4) (Fig. 2). Further comparative analysis of the characteristics of these two subgroups (Table 5) found that patients with AFP ≥ 200 ng/ml had greater tumour burden at baseline as identified by BCLC stage C (*p* = 0.017), larger mean tumour size (*p* = 0.002), and macrovascular invasion (*p* = 0.035) compared to patients with AFP < 200 ng/ml following initial TACE. In comparison, patients with serum AFP < 200 ng/ml were less likely to have underlying HCV (*p* = 0.004) or HBV (0.05), higher rates of T2DM (*p* = 0.05) and lower inflammatory response following TACE with lower serum neutrophils (*p* = 0.001) and ALT (*p* = 0.03).

**Table 5:** Characteristics of patients following initial TACE with AFP ≥ 200 ng/ml vs. < 200 ng/ml

Repeat serum AFP subgroup ***n*** = 177					
Variable	n	AFP < 200 ng/ml	n	AFP ≥ 200 ng/ml	***P*** value
Pre-TACE, % (n)					
Caucasian	147	83.7% (123)	30	63.3% (19)	0.011
HCV	147	41.5% (61)	30	70% (21)	0.004
HBV	147	17.7% (26)	30	33.3% (10)	0.05
T2DM	147	34.7% (51)	30	16.7% (5)	0.05
BCLC stage C	147	2% (3)	30	13.3% (4)	0.017
Macrovascular invasion	147	1.4% (2)	30	10% (3)	0.035
tumour size, (cm), mean (SD)	147	3.84 (2.55)	30	5.64 (3.89)	0.002
Post initial TACE, median (IQR)					
Neutrophils, x10^9/L	91	2.9 [2.3-3.72]	23	4.2 [2.9-5.1]	0.001
ALT, U/L	146	38.5 [24-72]	29	65 [34-100]	0.03
Na, mmol/L	145	138 [136-140]	28	137 [135-139]	0.048

To further explore the relationship between OS and AFP levels, we analysed the survival of patients according to the pattern of change in serum AFP levels < and ≥ 200 ng/ml pre- and post-initial TACE. Notably, the median survival of patients with an AFP ≥ 200 ng/ml both pre- and post-initial was similar to subjects whose level fell below 200 ng/ml after TACE (18.2 vs 19.9 months) (Table 6). In comparison, the median OS of patients whose AFP remained < 200 ng/ml both pre- and post-initial TACE was significantly higher at 34.7 months compared to the groups whose AFP level was either ≥ 200 ng/ml at baseline or increased to ≥ 200 ng/ml after initial TACE (*p* = 0.0001) (Table 6).

**Table 6:** Median survival time by AFP level of 200 ng/ml at baseline and post initial TACE

AFP groups	n	Median Survival time, months (IQR)	***P*** value
AFP0 < 200 and AFP1 < 200	135	34.7 (19.9 - 56.1)	0.0001
AFP0 < 200 and AFP1 ≥ 200	5	19.4 (14.2 - 29.6)	
AFP0 ≥ 200 and AFP1 < 200	12	19.9 (13.3 - 27.7)	
AFP0 ≥ 200 and AFP1 ≥ 200	25	18.2 (9.2 - 26.1)	

## Discussion

TACE is an effective treatment in eligible patients with uHCC, however the majority of patients treated with TACE will require repeat therapy due to a partial response or tumour recurrence. Treatment outcomes after TACE are influenced by both the severity of underlying liver dysfunction and tumour burden [1–3, 32], and as such the indications and criteria for repeat TACE remain variably defined and adopted in International guidelines [5, 26, 33, 34]. We therefore explored the factors associated with overall survival in patients having repeat TACE in a real-world multicenter cohort focusing particularly on the prognostic role of serum AFP level.

Serum AFP as a marker of tumour burden has been previously proposed as a prognostic marker in patients undergoing TACE for uHCC [35–38]. However, the prognostic role of serial changes in serum AFP levels following TACE has been controversial as not all HCC produce AFP, and false positive results not infrequently occur such as in active viral hepatitis [36, 39–41]. Consequently, a wide variety of serum cut off values have been postulated ranging from 20 to 400 ng/ml, as well as variations in serum AFP response ranging from 20 to 50% based on the AUROC of the derivation cohort [42–48]. Notably, application of these percentage change values in pre and post treatment AFP had no significant prognostic effect on OS in our cohort, and may reflect the inherent differences in our characteristics at baseline and also the inability of the delta value to capture the wide variations in serum AFP levels associated with HCC [49].

In contrast several recent studies including a meta-analysis have demonstrated a significant association of specific serum levels of AFP with HCC treatment outcomes including treatment response and overall survival [43, 50, 51]. In a recent prognostic model Wang et al. found the serum AFP of 400 ng/ml was a useful cut off value in a population with predominantly HBV related liver disease [38, 45]. They found the AFP response following TACE independently associated with prognosis in BCLC stage B patients, however further analysis regarding combination therapy with other treatments such as ablation or systemic therapies was not available.

When applied to our cohort, we found serum AFP cut off level of 200 ng/ml had the greatest stratification compared to an AFP < 20 ng/ml that was seen in 135 (52%) and AFP < 400 ng/ml in 215 (83%) of patients. This AFP value has the potential advantage over the lower cut off value of < 20 ng/ml in being less likely to include those with an elevated level due to active liver disease such as such as with chronic viral hepatitis [36, 41]. A serum AFP < 200 ng/ml was a significant prognostic marker both pre and post initial TACE associated with better overall survival outcomes (Fig. 2). Patients that maintained an AFP < 200 ng/ml at baseline and following initial TACE had a significantly better survival outcome (*p* = 0.0001) compared to patients that had a higher baseline AFP ≥2 00 ng/ml regardless of a post treatment change in levels. The poorer prognosis in patients with AFP ≥ 200 ng/ml following TACE may relate to the greater tumour burden at baseline including tumour size (*p* = 0.002) and macrovascular invasion (*p* = 0.035) along with greater inflammatory response to treatment with higher serum ALT (*p* = 0.03) and neutrophil count (*p* = 0.001). Further detailed analysis of serum AFP pre and post TACE is limited by small number of patients in each subgroup.

Our results are consistent with recent updates in international guidelines [26, 52] that have endorsed the revised cut off of 200 ng/ml from the previously used 400 ng/ml due to superior sensitivity and specificity and nearly 99% positive predictive value [36, 49, 53]. In particular explant studies have demonstrated higher AFP levels ≥200 ng/ml are associated with higher risk of both microvascular and macrovascular invasion, along with poorly differentiated tumours as was demonstrated in our analysis (Table 3) [21, 54]. However, the reduced availability of repeat AFP data in 61% of our overall cohort limits the generalisability of our findings particularly in cases of non-AFP producing HCC.

Along with AFP level, we found like others on multivariate analysis that markers of both hepatic reserve and tumour burden are key prognostic markers being associated with poorer survival following repeat TACE [14, 55–59]. In particular, decreasing liver reserve had the greatest impact on mortality in our cohort with post TACE liver failure and decompensation associated with the highest risk of reduced survival [HR 4.50, (95% CI 1.86–10.89), *p* = 0.001], (*p* = 0.001). Although this occurred in only 1% of our patients and is generally thought to be low in incidence (2–7%) [60–62], the frequency of liver decompensation may be as high as 18% following TACE depending on the definition used [30, 63–65]. We also found like others that decline in liver function as measured by higher serum bilirubin prior to repeat TACE was associated with reduced survival [66–68]. This again highlights the prognostic importance of liver reserve following TACE because discontinuation of TACE due to liver decompensation and/or biochemical decline carries a poorer prognosis than when it is due to radiological progression. Indeed, most patients with significant hyperbilirubinaemia are unsuitable for or intolerant of further therapies such as systemic therapy and are managed with best supportive care [69–71].

In addition, the number of tumour nodules prior to initial TACE was a significant and independent prognostic marker following repeat TACE in our cohort being associated with a higher risk of mortality. This is similar to the findings of several previous studies [57, 72, 73]. Furthermore, greater tumour size at baseline was significantly associated with higher serum AFP ≥ 200 ng/ml following initial TACE which was one of the key prognostic determinants associated with lower OS on multivariate analysis (Table 4).

As noted above an important limitation of our study was the reduced availability of serial AFP levels before and after the initial TACE to explore the relationship with survival further. However, there was only minimal differences in the characteristics in those without follow up AFP levels, with lower rates of HCV infection (*p* = 0.024), higher NAFLD (*p* = 0.03), and higher proportion of single HCC (p = 0.03) (data not shown). Other study limitations include the retrospective analysis of data with resultant variations in the timing of serum collection pre and post TACE as well as variations in on-site specific protocols, and this may impact on the interpretation of results that are influenced by post treatment hepatic dysfunction and inflammation such as serum AFP [36, 41].

Our study also includes patients undergoing combination therapies with TACE and a small number of BCLC stage C (4%) patients that underwent TACE outside of current guidelines, consistent with contemporary real-world studies analysing global patterns of TACE utilisation [15, 16]. Despite these limitations this is a large study analysing prognostic factors in patients undergoing repeat TACE on demand for uHCC over a significant period of 6 years across six large tertiary referral centres. As such these results have greater clinical relevancy to current clinical practice as the data incorporates variations in patient selection, TACE type (conventional or DEB), and technique (selective or super selective).

## Conclusions

Patient selection for repeat TACE requires a careful balance between the risk of complications, and benefits, with evaluation of both pre and post treatment factors associated with poor outcomes. These include higher baseline tumour burden, and decline in liver reserve. In particular, serum AFP is a useful prognostic marker for risk stratification, with an AFP ≥ 200 ng/ml post initial TACE associated with significantly poorer outcomes in those undergoing repeat TACE.

## Data Availability

The datasets generated and analysed during the current study are available from the corresponding author on reasonable request.

## Appendix

### mRECIST Definition [31]

Radiological response will be defined according to standard EASL criteria using modified RECIST (mRECIST) criteria as assessed by triphasic CT scan and/or MRI scan and include the following*:*

Complete response (CR): Disappearance of any intra-tumoural arterial enhancement in all target lesions

Partial response (PR): At least a 30% decrease in the sum of the diameters of viable (enhancement in the arterial phase) target lesions

Stable disease (SD): Any cases that do not qualify for either PR or progressive disease (PD)

Progressive disease (PD): An increase of at least 20% in the sum of the diameters of viable (enhancing) target lesions, taking as reference the smallest sum of the diameters of viable (enhancing) target lesions recorded since treatment started.

## Abbreviations

AFP: Alpha-fetoprotein
BCLC: Barcelona Clinic Liver Cancer
CI: Confidence interval
CPS: Child Pugh score
CR: Complete response
DEB: Drug-eluting bead
ECOG: Eastern Cooperative Oncology Group
EHS: Extra hepatic spread
HBV: Hepatitis B virus
HCC: Hepatocellular carcinoma
HCV: Hepatitis C virus
HR: Hazard ratio
INR: International normalised ration
KM: Kaplan-Meier
MDT: Multidisciplinary team
mRECIST: Modified Response Evaluation Criteria in Solid Tumours
NAFLD: Non-alcoholic fatty liver disease
OS: Overall survival
PD: Progressive disease
PEI: Percutaneous ethanol injection
PR: Partial response
PTS: Post TACE syndrome
RCT: Randomised controlled trial
SD: Stable disease
SIRT: Selective internal radiation therapy
TACE: Transarterial chemoembolisation
MVI: Macrovascular invasion
